# iTRAQ-proteomics and bioinformatics analyses of mammary tissue from cows with clinical mastitis due to natural infection with *Staphylococci aureus*

**DOI:** 10.1186/1471-2164-15-839

**Published:** 2014-10-02

**Authors:** Jinming Huang, Guojing Luo, Zijing Zhang, Xiuge Wang, Zhihua Ju, Chao Qi, Yan Zhang, Changfa Wang, Rongling Li, Jianbin Li, Weijun Yin, Yinxue Xu, Sonia J Moisá, Juan J Loor, Jifeng Zhong

**Affiliations:** Dairy Cattle Research Center, Shandong Academy of Agricultural Sciences, No.159 North of Industry Road, Jinan, Shandong 250131 China; College of Animal Science and Technology, Nanjing Agricultural University, Nanjing, 210095 China; Mammalian NutriPhysioGenomics, Department of Animal Sciences, University of Illinois, Urbana, IL 61801 USA; Division of Nutritional Sciences, University of Illinois, Urbana, IL 61801 USA; Department of Animal Sciences, University of Illinois, Urbana, IL 61801 USA

**Keywords:** iTRAQ, Proteomics, COL1A1, ITIH4, Dairy cow, Mastitis

## Abstract

**Background:**

Proteomics and bioinformatics may help us better understand the biological adaptations occurring during bovine mastitis. This systems approach also could help identify biomarkers for monitoring clinical and subclinical mastitis. The aim of the present study was to use isobaric tags for relative and absolute quantification (iTRAQ) to screen potential proteins associated with mastitis at late infectious stage.

**Results:**

Healthy and mastitic cows’ mammary gland tissues were analyzed using iTRAQ combined with two-dimensional liquid chromatography-tandem mass spectrometry (2D-LC-MS/MS). Bioinformatics analyses of differentially expressed proteins were performed by means of Gene Ontology, metabolic pathways, transcriptional regulation networks using Blast2GO software, the Dynamic Impact Approach and Ingenuity Pathway Analysis. At a false discovery rate of 5%, a total of 768 proteins were identified from 6,499 peptides, which were matched with 15,879 spectra. Compared with healthy mammary gland tissue, 36 proteins were significantly up-regulated (>1.5-fold) while 19 were significantly down-regulated (<0.67-fold) in response to mastitis due to natural infections with *Staphylococci aureus*. Up-regulation of collagen, type I, alpha 1 (COL1A1) and inter-alpha (Globulin) inhibitor H4 (ITIH4) in the mastitis-infected tissue was confirmed by Western blotting and Immunohistochemistry.

**Conclusion:**

This paper is the first to show the protein expression in the late response to a mastitic pathogen, thus, revealing mechanisms associated with host tissue damage. The bioinformatics analyses highlighted the effects of mastitis on proteins such as collagen, fibrinogen, fibronectin, casein alpha and heparan sulfate proteoglycan 2. Our findings provide additional clues for further studies of candidate genes for mastitis susceptibility. The up-regulated expression of COL1A1 and ITIH4 in the mastitic mammary gland may be associated with tissue damage and repair during late stages of infection.

**Electronic supplementary material:**

The online version of this article (doi:10.1186/1471-2164-15-839) contains supplementary material, which is available to authorized users.

## Background

Mastitis, an inflammation of the mammary gland, remains the most prevalent disease and the largest economic loss in dairy cattle. The economic impact of mastitis on the U.S dairy industry in 1976 was estimated at $1.294 billion and increased up to $2 billion in the year of 2009 [1]. A large number of microorganisms, most of which are bacteria, have been reported to cause bovine mastitis. Worldwide, however, the most common udder pathogens isolated from the clinical cases are *Staphylococci aureus* (*S. aureus*), *Streptococcus dysgalactiae* and *Escherichia coli*. The *S. aureus* is considered as a contagious mastitis-causing pathogen and remains an important mastitis pathogen in most countries [2]. The frequency of mastitis in the dairy cow population could potentially be decreased by breeding for cows with better ability to resist udder disease. Therefore, identifying specific genes involved in the susceptibility or resistance to mastitis could lead to new approaches for mastitis control through genetic selection [3].

Mastitis involves a complex set of interactions between an invading pathogen and immune systems of the host. Proteomics and the associated bioinformatics are considered as complimentary tools for the study the dynamic interactions between the immune system and pathogens [4]. Most proteomic studies on mastitis conducted to date have been performed using two-dimensional electrophoresis (2-DE) and liquid chromatography (LC) coupled with tandem mass spectrometry (MS) methods [5–8] and using milk, serum or somatic cells. For instance, differential expression analysis of the whey from both mastitic and non-mastitic milk revealed a series of proteins including acute phase proteins (APP), lactotransferrin and immunoglobulins that present a marked alternation during infection [6, 7].

Several studies have reported proteomics profiles in milk and serum of cows infected with different pathogens [9, 10]. For instance, a total of 2971 milk proteins were identified and more than 300 milk proteins associated with host defense were identified in *S. aureus* infected and normal milk using the isobaric tag for relative and absolute quantification (iTRAQ) method [10]. Differentially expressed milk proteins at 2 and 14 days post-intramammary infection with different *S. aureus* strains have also been identified by 2DE [9]. Forty-seven peptide biomarkers of milk for the diagnosis of mastitis were discovered using capillary electrophoresis and mass spectroscopy [11].

The transcriptional response of the mammary gland to mastitis infected different pathogens has been reported in several microarray studies [12, 13], while information at the proteome level in mammary gland tissue is still limited particularly in animals with natural infections. Persistence of pathogen infections through late stages can worsen damage of the mammary gland and result in milk lost, or leave necrotic tissue and important injures [14]. Therefore, the investigation of changes in the protein expression upon the onset, progression and late onset of mastitis is crucial for providing a full picture of the events triggered by this disease. The exact quantification of differentially expressed proteins has proven difficult with gel-based approaches. Nevertheless, to date, there are no proteomic studies aimed at investigating the susceptibility to *S. aureus*-associated mastitis in dairy cows.

Here we present an exploration of the mastitis-induced changes in the proteome of mammary gland tissue using the iTRAQ system. This approach allows the simultaneous identification and quantitative comparison of peptides by measuring peak intensities of reporter ions in tandem mass spectrometry (MS/MS) spectra [15]. Differentially expressed proteins were used for bioinformatics analyses via the Gene Ontology (GO), Dynamic Impact Approach (DIA) [16] and the Ingenuity Pathway Analysis (IPA).

## Results

### Protein spectrum and GO analysis of mammary gland tissue of dairy cows

**Figure 1:**
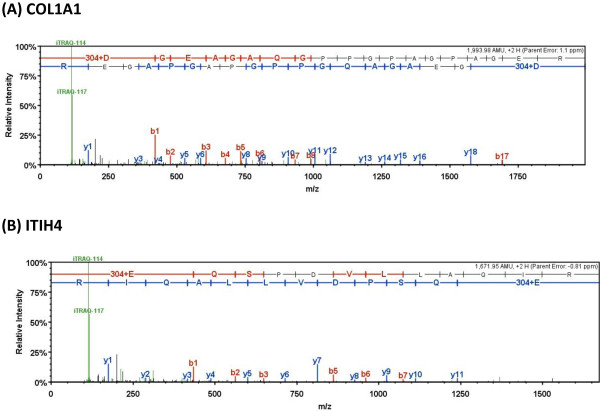
**MS/MS spectrum for the relative quantity of two representative proteins (a) COL1A1 (Accession: tr|F1MGW0|F1MGW0_BOVIN); (b) ITIH4 (Accession: tr|Q5EA67|Q5EA67_BOVIN).** The two lines on the top of the peak figure correspond to the peptides sequence (the first line is the positive sequence, the second line is a reverse sequence); Amino acid with bold letters present detected sequence. Amino acid + digital present the decorated aa, while the number shows the quality deviation occurred, such as + 304 denotes the ITRAQ modification is introduced by the ITRAQ reagent. Horizontal axis is the value of m/z and the vertical axis is the normalized intensity. B1, b2, b3,… Y1, y2, y3… correspond to the peak of the detected b and y ions. The peaks corresponding to ITRAQ–114 and ITRAQ-117 mark these 2 pool samples. The intensity of two peaks was used for the quantitative of protein. The peak figure is done by Scaffold software at http://www.proteomesoftware.com/.

In the present study, iTRAQ technology in combination with LC-ESI-MS/MS was applied to investigate differentially expressed proteins in the healthy and mastitis-infected mammary glands of dairy cows. A total of 768 proteins were identified from 6499 peptides according to the standard of protein identification (Additional file 1). Of which, 35.42% proteins were identified with one peptide and 45.31% proteins with 3 to 177 peptides. COL1A1, collagen alpha-1(I) chain possesses the most of peptides. There are 177 peptides covering the 52.4% protein sequence with above 95% confidence. Several proteins like lactoferrin, MYH11, alpha-S1-casein, COL3A1, serum albumin, COL6A3 and COL1A2 were identified with over 50 peptides. Figure 1 shows the relative expression of two proteins (COL1A1 and ITIH4) in the healthy and mastitis-infected mammary gland tissues which was calculated as a ratio by comparing the intensity of the iTRAQ-114 and iTRAQ-117 after normalization.

Expressed proteins in mammary gland tissues of cows were classified by the DAVID online program (http://david.abcc.ncifcrf.gov/). These proteins were clustered into 178 clusters including cytoskeletal protein binding, signal peptides, etc. Gene functional annotation analysis indicated that thirty proteins such as, CD14, serum amyloid 3, casein alpha-S2, cathelicidin 2, cathelicidin 6, cathelicidin antimicrobial peptide, complement component 3 (C3), complement component 6 (C6), complement factor B were involved in defense, immune and inflammatory reactions (Table 1). Six hundred genes participated in 32 pathways like ECM-receptor interaction (17 genes), complement and coagulation cascades (16 genes), and focal adhesion (30 genes) KEGG pathways (Additional file 2: Table S1).

**Table 1:** **Proteins involved in the defense, immune and inflammatory reactions by DAVID analysis**

Accession	Protein names (full)	Official gene name	Peptides (95%)	Fold-change (114:117)	P value 114:117
tr|Q06AV9	Monocyte differentiation antigen CD14	CD14	2	1.20	0.77
tr|F1MQF6	Apoptosis-associated speck-like protein containing a CARD	PYCARD	1		
sp|P79105	Protein S100-A12	S100A12	6	11.11	0.00
sp|Q0VCA5	SAM domain and HD domain-containing protein 1	SAMHD1	2	0.85	0.71
tr|F5CC79	Beta-1,4-galactosyltransferase I	B4GALT1	2	0.89	0.22
tr|B0JYN6	Alpha-2-HS-glycoprotein	AHSG	3	0.93	0.49
sp|P81644	Apolipoprotein A-II	APOA2	1	0.74	0.47
tr|F1N3Q7	Apolipoprotein A-IV	APOA4	5	0.24	0.01
sp|P02663	Alpha-S2-casein	CSN1S2	25	0.19	0.00
tr|B9TUB8	Cathelicidin 2	CATHL2 BAC5	2	1.06	1.00
tr|B9UKM3	Cathelicidin 6	CATHL6	4	1.41	0.26
sp|P19661	Cathelicidin-3	CATHL3 BAC7	2	1.09	1.00
sp|Q2HJ57	Coactosin-like protein	COTL1	3	1.05	0.32
sp|P00735	Prothrombin	F2	4	2.17	0.31
sp|Q2UVX4	Complement C3	C3	30	0.63	0.06
tr|F1MM86	complement component C6 precursor	C6	1	1.18	0.76
sp|P81187	Complement factor B	CFB	4	1.05	0.25
tr|B8Y9T0	Cumulus cell-specific fibronectin 1 transcript variant	FN1	14	7.14	0.00
sp|Q28085	Complement factor H	CFH	3	1.06	0.64
tr|Q5EA67	Inter-alpha (Globulin) inhibitor H4	ITIH4	10	4.55	0.00
tr|F2X043	Lactoperoxidase	LPO	11	0.97	0.30
tr|F1MNN7	Lipopolysaccharide-binding protein	LBP	1	1.10	0.51
sp|Q1ZZU7	Macrophage migration inhibitory factor	MIF	7	0.96	0.41
tr|B5T254	Peptidoglycan recognition protein 1	PGLYRP1	5	1.05	0.76
tr|F4YD22	Peroxiredoxin 1	PRDX1	2	1.00	1.00
sp|Q9BGI3	Peroxiredoxin-2	PRDX2	5	0.99	0.83
tr|Q8SQ28	Amyloid protein A	Saa3	5	2.86	0.15
sp|P33046	Cathelicidin-4	CATHL4	7	1.85	0.30
tr|F1N3A1	Thrombospondin-1	THBS1	3	3.03	0.04
tr|F1MIN1	Voltage-dependent anion-selective channel protein 1	VDAC1	2	0.92	0.69

### Identification and pathway analysis of the differential proteins between the healthy and mastitic cows’ mammary gland tissues

Basing on the standard of the differentially expressed proteins, 36 proteins were significantly differentially up-regulated (>1.5-fold) while 19 proteins were significantly down-regulated (<0.67-fold) in mastitis-infected cow group’s proteins labeled ITRAQ-114 when compared with that of the healthy group’s proteins labeled ITRAQ −117 (Table 2).

**Table 2:** **Differentially expressed proteins between the healthy and mastitis-infected cows’ mammary gland tissues**

Accession	Protein names	Official gene name	Peptides (95%)	Fold-change (114:117)	P value 114:117
***Up-regulated proteins in the mastitis group***					
tr|F1MGW0	Collagen alpha-1(I) chain	COL1A1	177	3.70	0.0001
tr|E1BB91	Collagen alpha-3(VI) chain	COL6A3	100	2.99	0.0000
sp|P02465	Collagen alpha-2(I) chain	COL1A2	100	3.56	0.0002
sp|P02769	Serum albumin OS	ALB	89	2.27	0.0000
tr|F1N169	Filamin-A	FLNA	51	2.61	0.0000
tr|F1MQ37	myosin-9	MYH9	49	2.23	0.0010
tr|F1N4K8	ENSBTAG00000002278	FBN1	31	3.19	0.0000
tr|E1BI98	Collagen alpha-1(VI) chain	COL6A1	30	2.58	0.0026
tr|D4QBF3	Hemoglobin beta	HBB	25	3.50	0.0000
tr|F1MKG2	collagen alpha-2(VI) chain precursor	COL6A2	22	2.31	0.0032
sp|P02676	Fibrinogen beta chain	FGB	22	11.48	0.0000
tr|A5PJE3	Fibrinogen alpha chain	FGA	22	8.87	0.0000
sp|O62654	Desmin	DES	20	7.24	0.0000
sp|Q7SIH1	Alpha-2-macroglobulin	A2M	18	3.50	0.0000
tr|F1MER7	Basement membrane-specific heparan sulfate	HSPG2	17	1.77	0.0180
sp|Q9TTE2	Decorin	DCN	17	2.29	0.0109
sp|Q27991	Myosin-10	MYH10	16	3.10	0.0386
sp|Q9TTE1	Serpin A3-1	SERPINA3-1	15	3.34	0.0216
tr|B0FZM4	Myosin light chain 6	MYL6	15	1.67	0.0106
tr|B8Y9T0	Cumulus cell-specific fibronectin 1 transcript variant	FN1	14	6.92	0.0000
tr|Q3SZZ9	Fibrinogen gamma-B chain	FGG	14	16.75	0.0000
tr|Q5EA67	Inter-alpha (Globulin) inhibitor H4	ITIH4	10	4.49	0.0000
tr|F1MIQ8	Biglycan	BGN	9	2.54	0.0133
sp|P79134	Annexin A6	ANXA6	8	2.00	0.0233
sp|P01967	Hemoglobin subunit alpha-1	HBA1	8	4.41	0.0020
sp|Q2TBU0	Haptoglobin	HP	7	5.45	0.0020
sp|P79105	Protein S100-A12	S100A12	6	11.17	0.0002
sp|Q2KJH6	Serpin H1	SERPINH1	6	3.05	0.0040
tr|F1MHS5	Protein S100-A9	S100A9	6	5.01	0.0097
tr|E1B6Z6	Neutrophil gelatinase-associated lipocalin	LCN2	5	3.56	0.0049
tr|F1N076	ENSBTAG00000012164	CP	4	5.81	0.0129
sp|P51176	Protein-glutamine gamma-glutamyltransferase 2	TGM2	4	8.32	0.0069
sp|P28782	Protein S100-A8	S100A8	4	5.01	0.0499
tr|A3KN02	HIST1H1C protein	HIST1H1C	3	1.69	0.0011
tr|F1N3A1|	Thrombospondin-1	THBS1	3	3.08	0.0434
tr|F1MTV8	ENSBTAG00000047345	ENSBTAG00000047345	2	21.68	0.0455
***Down-regulated proteins in the mastitis group***					
sp|P02662	Alpha-S1-casein	CSN1S1	65	0.10	0.0011
tr|F1N2D9	Uncharacterized protein	F1N2D9	45	0.48	0.0000
tr|B5B0D4	Heat shock protein 90 kDa beta member 1	HSP90B1	32	0.12	0.0000
tr|Q9N273	Kappa-casein (Fragment)	CSN3	27	0.17	0.0018
sp|P02663	Alpha-S2-casein	CSN1S2	25	0.19	0.0001
tr|F1MU12	Keratin, type II cytoskeletal 8	KRT8	24	0.27	0.0000
sp|P08728	Keratin, type I cytoskeletal 19	KRT19	18	0.27	0.0024
tr|F1MLK0	Isocitrate dehydrogenase [NAD] regulatory subunit 1, mitochondrial	IDH1	14	0.52	0.0036
sp|P17248	Tryptophanyl-tRNA synthetase, cytoplasmic	WARS	10	0.41	0.0019
sp|Q5E946	Protein DJ-1	PARK7	7	0.21	0.0096
sp|P00727-2	Isoform 2 of Cytosol aminopeptidase	LAP3	7	0.58	0.0465
sp|P20000	Aldehyde dehydrogenase, mitochondrial	ALDH2	7	0.43	0.0337
sp|P81265	Polymeric immunoglobulin receptor	PIGR	6	0.20	0.0081
sp|P42899	60S acidic ribosomal protein P2	RPLP2	5	0.35	0.0069
tr|F1N3Q7	Apolipoprotein A-IV	APOA4	5	0.24	0.0129
sp|P51977	Retinal dehydrogenase 1	ALDH1A1	5	0.34	0.0146
tr|F1MDK4	Ethylmalonyl-CoA decarboxylase	ECHDC1	5	0.19	0.0108
tr|F1MZQ4	Butyrophilin, subfamily 1, member A1	BTN1A1	3	0.17	0.0052
tr|C4T8B4	C-reactive protein	CRP	3	0.25	0.0027

In term of GO database, the differentially expressed proteins were divided into CC, MF and BP categories. The top 5 GO terms for CC with the minimal p values were extracellular region, extracellular region part, extracellular space, extracellular matrix and extracellular matrix part (Additional file 3: Figure S1). The top 5 GO terms for MF were growth factor binding, cell surface binding, metal ion binding, motor activity and cation binding (Additional file 4: Figure S2). The top 12 GO terms with a p-value <0.01 for MF were response to stress, response to stimulus, skeletal system development, response to inorganic substance, gas transport, peptide cross-linking, skin development, protein heterooligomerization, extracellular structure organization, response to steroid hormone stimulus, response to chemical stimulus and defense response (Additional file 5: Figure S3; Additional file 2: Table S2). For example, 10 proteins [alpha-S2-casein (CSN1S2), C-reactive protein (CRP), neutrophil gelatinase-associated lipocalin (LCN2), thrombospondin-1 (THBS1), protein S100-A9 (S100A9), ENSBTAG00000047345, inter-alpha (Globulin) inhibitor H4 (ITIH4), protein S100-A12 (S100A12), protein S100-A8 (S100A8) and alpha-2-macroglobulin (A2M)] participated in the defense, immune and inflammatory responses (Additional file 2: Table S2). Another important protein was C3 with a p-value 0.06, involved also in the immune response (Table 1). Of 10 proteins, the expression of ITIH4 protein was increased by 4.55-fold in the mastitis group when compared with the control group. ITIH4 protein was enriched in the membrane, cell, intracellular and intracellular part terms for CC (Additional file 3: Figure S1); it was also enriched in the nonpeptidase/peptidase/enzyme inhibitor/regulator activity terms for MF (Additional file 4: Figure S2); it is related with the response to stress/stimulus, defense response, acute or inflammatory response, amine/carbohydrate/macromolecule/nitrogen compound metabolic processes terms for BP (Additional file 5: Figure S3). Moreover, according to the BP classification, the differential protein COL1A1 with the most of peptides were involved in 91 GO terms, such as response to stimulus, extracellular structure organization, cellular component assembly, etc. (Additional file 2: Table S2; Additional file 5: Figure S3). COL1A1 enriched in the 12 GO terms for CC, for example, extracellular region part, fibrillar collagen and intracellular part (Additional file 3: Figure S1). Basing on the MF classification, COL1A1 were also related with 4 GO terms including growth factor binding, structural molecule activity, proteins binding and binding (Additional file 4: Figure S2).

Pathway enrichment analysis showed that 51 differential proteins participated in 62 pathways. Among them, 8 proteins (COL1A1, CO1A2, COL6A3, COL6A2, COL6A1, FN1, HSPG2 and THBS1) were enriched in the ECM-receptor interaction pathway that serves an important role in tissue and organ morphogenesis and in the maintenance of cell and tissue structure and function, 6 proteins (A2M, FGB, FGA, FGG, HP and LCN2) participated in the complement coagulation cascades pathway. The ITIH4 and FLNA proteins took part in the MAPK signaling pathway. Two proteins FGG and HP upregulated by 16.75- and 5.45- fold in mastitis-infected cow’s tissues were involved in *S. aureus* infection pathway (Table 2 and Additional file 2: Table S3).

DIA analysis (Additional file 6) revealed that complement and coagulation cascade was the KEGG pathway with the highest impact value in which fibrinogen complex (GO:0005577), platelet activation (GO:0030168) and protein binding, bridging (GO:0030674) were the significantly impacted GO Terms.

Focal adhesion, amoebiasis, protein digestion and absorption, and ECM-receptor interaction pathways were also significantly impacted in the DIA analysis. These pathways had in common COL1A1, COL1A2 and FN1 proteins, with activation of skin morphogenesis (GO: 0043589), cartilage development involved in endochondral bone morphogenesis (GO: 0060351), collagen fibril organization (GO: 0030199), platelet activation (GO: 0030168) and protein binding, bridging (GO: 0030674) GO Terms (Additional file 6).

Some GO Terms related to a specific protein but not to any pathway with a high impact and inhibition were casein, alpha/beta (IPR001588) for alpha-S2B-casein (CSN1S2), cartilage development involved in endochondral bone morphogenesis (GO:0060351) and endochondral ossification (GO:0001958) for HSPG2 and CSN1S1 proteins, platelet activation (GO:0030168) for ALB, and oxidoreductase activity, acting on the aldehyde or oxo group of donors, NAD or NADP as acceptor (GO:0016650/GO:0016903) for ALDH1A1 (Additional file 6).

### Validation of two candidate proteins COL1A1 and ITIH4

**Figure 2:**
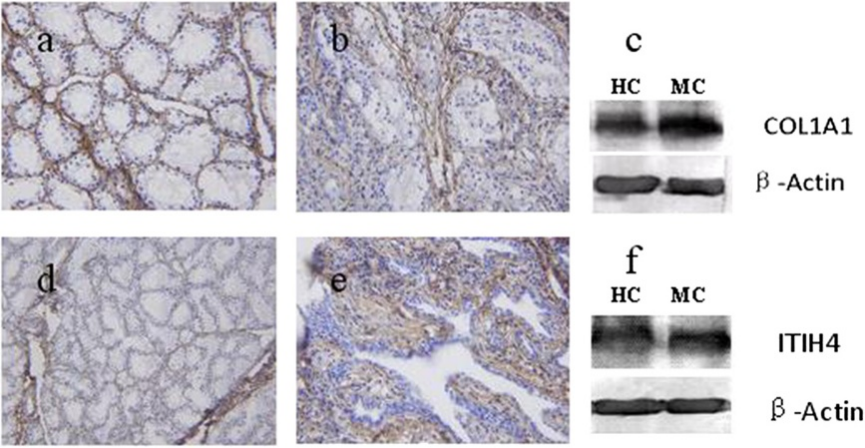
**Expression and localization of bovine COL1A1 and ITIH4 proteins. a**, **b**: The localization and expression of COL1A1 in the healthy cow (HC) and mastitis cow’s (MC) mammary gland tissues by immunohistochemical staining. Brown indicates the positive COL1A1 protein expression; blue presents the negative. **d**, **e**: The localization and expression of ITIH4 in the HC and MC group’s mammary gland tissues by immunohistochemical staining. Brown indicates the positive ITIH4 protein; blue presents the negative. **c**, **f**: The results of Western blotting reveal the significant increase of COL1A1 and ITIH4 proteins in MC group compared to HC group (p < 0.05).

Among the identified proteins, two proteins COL1A1 and ITIH4 were considered as interesting protein for further studies. Firstly, the relative expressions of COL1A1 (~50 kDa) and ITIH4 (~100 kDa) in the healthy and mastitis-infected cows’ mammary gland tissues were investigated by the Western blotting. The results showed that the expression of two proteins significantly increased in the mastitis-infected group when compared with the healthy control group (P < 0.05). These two proteins were also detected in the mammary gland tissue of two groups by the immunohistochemical staining method (Figure 2). However, it should be considered that the apparent difference of COL1A1 and ITIH4 observed by Western blotting and IHC methods were smaller than that seen by iTRAQ (COL1A1, 177 peptides; ITIH4, 10 peptides), although consistent with the observations made by iTRAQ, respectively, might be due to differences in specificity of the commercially available antibodies used in this work, which are not specific for bovine proteins. In the present study, for example, several collagen alpha family proteins (COL1A1, COL1A2, COL6A1, COL6A2 and COL6A3) present the similar differentially expressed tendency from 2.31-fold to 3.7-fold (Table 2). Therefore, the quantitative conclusions should be drawn only by means of quantitative proteomic approaches or alternative quantitative assays.

## Discussion

In this study, we performed a comprehensive evaluation of proteomic profile in the mammary glands of cows infected naturally by *S. aureus*, providing new data on the *in vivo* events occurring in the lactating mammary epithelium during persistent infection. Subsequently, GO enrichment and pathway analyses of proteins in combination with western blotting and immunohistochemistry detection for proteins of interest were focused on the consistent results involved in the immune, inflammatory and defense response for mastitis infection.

Generally, host defense against microbial pathogens requires appropriate coordination of multiple signaling pathways. These pathways are triggered by innate immune recognition of microbial molecules, and initiate an inflammatory cascade that involved recruitment of leukocytes to the site of infection, activation of antimicrobial effector mechanisms and induction of an adaptive immune response [17]. In the present study, we found the global expressed and differentially expressed proteins participated in respective 32 and 62 pathways including MAPK, ECM-receptor interaction, complement and coagulation cascades, and focal adhesion KEGG pathways (Additional file 2: Table S1 and Table S3). MAPK play key roles in activating host innate immune responses and are frequent targets of pathogenic effectors in animal systems [18]. The complement and coagulation cascades are connected to the immune system [19]. Cells of the immune system are activated by soluble chemokine signals and must migrate through endothelial cell or solid tissue barriers to reach sites of inflammation or infection during the progress of performing host-defense functions. Adhesive interactions of immune cells with endothelium, extracellular matrix components, and cells of solid organs are the critical control point of the immune response. Both the soluble chemokine and cell adhesion receptor-mediated migration signals must converge on common intracellular targets to engage the cell migration machinery [20].

Specifically, in this study, several differentially expressed proteins, such as fibrinogen proteins (FGA, FGB and FGG), complement proteins (C3 and C6) expressed in mammary gland participated in many pathways encompassing the MAPK, complement and coagulation cascades, focal adhesion, etc. The complement system plays a fundamental role in innate immunity in addition to enhancing adaptive immune responses and is therefore a primary line of defense against infection [21]. The beginning of the pathogenesis of mastitis is determined by the binding of *S. aureus* to platelets [22]. This binding is likely to be mediated in part by soluble bridging molecules (GO:0030674) like fibrinogen (GO:0005577) [23], that link the organism to the platelet surface (GO:0030168). Platelets play a key role in innate defenses against *Staphylococci* by releasing platelet microbicidal proteins that kill many *S. aureus* isolates. Platelet binding may be an important mechanism for host against mastitic infections, because platelets attached to damaged cell surfaces may serve as binding center for organisms circulating in the blood [24]. Moreover, clinical and subclinical mastitis is characterized by abnormalities in the coagulation process due to excessive fibrin deposition in microvasculature leading to clot formation [25]. C3 protein was numerically down-regulated in the mastitic cow’s mammary gland with the p value nearing significance. A2M protein belongs to the complement and coagulation cascades pathway, which has been reported that it is involved in the mastitis susceptibility in our previous publication [26, 27].

The combined use of iTRAQ labelling and LC ESI-MS/MS is a powerful tool for the proteomics identification of the dairy cow’s mammary gland tissues. In this study, only 768 proteins were detected in the cow’s mammary gland. Whereas, a transcriptome profiling study reveals more than 2,200 differential expressed genes (>1.5-fold) in the *Streptococcus uberis*-induced mastitis mammary gland when compared with noninfected control quarters [28]. Obviously, the number of proteins is by far, less than the amount of genes at the mRNAs level. The divergence might be caused by these factors, such as distinct response to different bacteria [29] and strain types [9], different infection ways (challenging and naturally occurring), different infection stages (early and late), translation regulation of mRNAs to proteins, or limitation of detection method. On the other hand, the contrasting data between proteomics and transcriptomics analysis, suggests that changes in protein profiles between the healthy and mastitis-infected cows’ mammary gland tissues provide information that is complementary to transcriptional profiling studies, revealing potentially crucial aspects of the mastitis infection response. Especially, when the mammary gland was at the state of fibrosis for the serious infection, expression of some proteins associated with metabolism would be shut down.

Three S100 calcium-binding proteins S100 calcium-binding protein A12 (S100A12), S100A8 and S100A9, are involved in the immune, defense and inflammatory responses, and have proinflammatory functions and direct antimicrobial effects [30, 31]. It has been reported that there is 5.2 fold to 49.1 fold up-regulation of S100A12 expression for three cows at the infection dose of 1 × 10^5^ *S. aureus* when compared with the intra-animal controls [32]. Furthermore, significantly increased S100A12 protein expression was detected in the milk whey from the infected udders in all three cows at 16 h post-infection [32]. In the present study, proteins S100A12, S100A8 and S100A9, were up-regulated by 11.17, 5.1 and 5.1 folds in the mastitis-infected mammary glands, which is consistent with the previous report, further demonstrating their roles in the immune response to pathogen infection.

Type I collagen, which is encoded by two subunits of COL1A1 and one subunit of COL1A2 wound together to form a triple-helix, of which the coordinated transcription rates ensure a 2:1 ratio. It is the most abundant protein in vertebrates and is the main structural protein of skin, bone, teeth, and tendon [33]. Moreover, it plays a major role in tissue and organ development, cell migration, proliferation, and differentiation, wound healing, tissue remodeling, and homeostasis and therefore it participates in the maintenance of organ morphology and function. Its excessive deposition results in the fibrosis of tissues [34].

Bovine mammary glands are composed of glandular tissue and connective tissue. It is easy to understand the most abundant expression of COL1A1. Our finding of type I collagen protein expression is consistent with the above result. During infection of the mammary glands, the tissue damage can initially be caused by bacteria and their products. Certain bacteria like *S. aureus* produce toxins that destroy cell membranes and damage milk-producing tissue, whereas other bacteria are able to invade and multiply within the bovine mammary epithelial cells. Then, more immune cells migrate into the mammary gland and harm the blood-milk barrier. Damage of the mammary epithelium gets worsen and it finally results in fibrosis [35]. Therefore, regulating the bovine COL1A1 expression for reducing tissue damage may be a cost-effective way to reduce the losses caused by mastitis.

Focal adhesion, amoebiasis, ECM receptor interacting and protein digestion and absorption pathways were highly impacted due to the activation of several proteins that might have a role in the conformational changes in the parenquimal epithelial cells due to *S. aureus* infection (Additional file 5). Heparan sulfate proteoglycan 2 (HSPG2) or perlecan blocks endothelial cell adhesion to fibronectin and type I collagen in the submucosal connective tissue from the parenquimal tissue of the infected mammary gland. Fibronectin plays a major role in cell adhesion, growth, migration and differentiation, and it is important for processes such as wound healing [36]. Altered fibronectin expression has been associated with fibrosis [37]. Milk obtained from cows with clinical mastitis possessed high counts of somatic cells and very high levels of protease activity which hydrolyzes casein [38]. This process was also detected by DIA analysis by a high impacted inhibition of caseins (IPR001588).

The acute-phase response occurs in animals and elevates APP as a consequence of infection, inflammation, or trauma. APP including serum amyloid A, haptoglobin, alpha-1-acid glycoprotein, alpha-1-proteinase inhibitor (alpha-1-antitrypsin) and ITIH4 have been shown to increase in different inflammatory processes, in both *in vivo* and *in vitro* models, and involved several inflammatory diseases, such as Crohn’s disease and ulcerative colitis [39–41]. ITIH4 protein is a 120KD glycoprotein, which is prone to be cleaved to produce fragments of different length, such as 100 kDa and 35 kDa fragment, which have been identified as the biomarkers for Down syndrome [41], amyotrophic lateral sclerosis [42], and cancer [43]. A report showed that all heifers responded to the mastitis with increased ITIH4 concentrations [44]. Severely infected heifers showed maximum ITIH4 concentrations at 72 h after bacterial injection. The concentration of the protein in serum was 6- to 12- fold higher than before the bacterial challenge. In the four animals with milder clinical mastitis, the protein exhibited only a three- to four-fold increase during the infection process and had reached a plateau by 48 h after the challenge. In both cases, the ITIH4 concentrations decreased to normal values about 2 weeks after the injection [44]. In this study, however, at the late stage of infection, the ITIH4 protein was first found to be significantly increased in the mammary gland tissues as compared to the control case. In this study, iTRAQ method is unable to differentiate cleaved fragments from whole protein. The result of western blotting implies that 100 kDa fragment of bovine ITIH4 plays an important role in the defense and immune responses to infection of mastitis.

## Conclusion

Proteomics analysis highlighted the effects of proteins like collagen, fibrinogen, fibronectin, casein alpha and heparan sulfate proteoglycan 2 among others in mammary gland tissues infected naturally by *S. aureus*. This study also established a protein database characteristic of the mammary response during late on-set of *S. aureus* naturally occurring mastitis. The iTRAQ technology and LC-MS/MS data generated, can be used as a reference for further research in this field.

Ours is the first to examine the late response to natural mastitis providing a ‘closer look’ into the mechanisms associated with host tissue damage. The differentially expressed proteins we discovered in this study, such as COL1A1 and ITIH4, may serve as potential genes for the susceptibility to mastitis in dairy cows.

## Methods

### Ethics statement

All experiments were carried out according to the Regulations for the Administration of Affairs Concerning Experimental Animals published by the Ministry of Science and Technology, China in 2004 and approved by the Animal Care and Use Committee from the Dairy Cattle Research Center, Shandong Academy of Agricultural Sciences, Shandong, P. R. China.

### Animals

Tissue samples were collected from mammary glands of first lactation Chinese Holstein cows (3.0- to 3.5-year-age) from the farm of the Dairy Cattle Research Center, Shandong Academy of Agricultural Sciences. The milk somatic cell count (SCC) of cows was measured by the instrument Fossomatic 5000 (Foss Electric, Denmark) once a month as part of the DHI program. Meanwhile pathogens in milk for each quarter were identified using cultures. First, the cows with the SCC > 5 × 10^6^ cells/mL and California mastitis test CMT score ≥2 were considered as the candidate individuals. In the end of the first month (The second milk sampling), we did not find any pathogen infection by culturing from milk samples. These candidate cows were treated with antibacterial drugs when they occurred elevated SCC along with *S. aureus* infection in milk cultures after two months (The third milk sampling). After approximately three months, the subclinical cows that developed into clinical mastitis as identified by abnormalities in the udder such as swelling, redness, hardness or pain clinical symptoms, had to be culled for the ineffective treatments. Thus, these cows with only the *S. aureus* infection were selected to provide the mastitic samples. The mastitic group used for this study was defined as those cows with *S. aureus* strain, designated zfb strain which is resistant to methicillin and could not produce lipase when compared with the ATCC 25923 standard strain. The colony morphology of *S. aureus* capsule was diffuse *in vivo* and *in vitro* when it was cultured in modified serum soft agar. At slaughter one tissue sample was collected then frozen in liquid nitrogen, and stored at −80°C for further protein analysis. Another fresh tissue sample was collected for pathogen identification described as our previous procedures [45, 46]. A cow was defined as healthy if the above clinical symptoms were not observed and the milk SCC was lower than 1 × 10^5^ cells/mL, and no pathogens were examined from the cow’s mammary tissues using culture and PCR methods [45]. After pathological evaluation, mammary gland tissues of 3 healthy and 3 mastitis-infected cows at a similar stage of lactation were categorized in the control and case groups. These samples were used for the iTRAQ, Western blotting and Immunohistochemistry analyses.

### Protein isolation and iTRAQ labeling

To each 0.5 g tissue sample was added 10% polyvinylpyrrolidone prior to grinding in liquid nitrogen. Then, the supernatant was transferred to another tube and five folds 10% chilled TCA acetone added for 2 hrs at −20°C. The precipitation was washed with chilled acetone for 30 mins, and the supernatant was discarded after centrifugation at 4°C, 20000 × g for 20 mins. The above steps were repeated 3 times. The pellet was dried in the air, then dissolved in 500 μl 0.5 M TEAB and sonicated at 200 Watts for 15 mins. Then it was centrifuge at 4, 30000 g for 20mins and the supernatant was quantified for protein using the GE Healthcare’s Quant kit (Code No. 80-6483-56).

A total of 100 μg protein for each sample was digested with Trypsin Gold (Promega) at a ratio of protein:trypsin of 20:1 at 37°C for 4 hrs. After trypsin digestion, the peptides were dried via vacuum centrifugation. The iTRAQ labeling was performed according to the manufacturer’s protocol (Applied Biosystems). Briefly, one unit iTRAQ reagent (Applied Biosystems) was thawed and reconstituted in 70 μL isopropanol. The healthy and mastitis groups were labeled with 117 and 114 iTRAQ reagent, respectively, by incubation at room temperature for 2 hrs. The labeled peptide samples were pooled and fractionated by strong cationic exchange (SCX) chromatography.

### Fractionation by SCX

For SCX chromatography using the LC-20AB HPLC Pump system (Shimadzu), the peptide from digestion was reconstituted with 4 mL buffer A (25 mM NaH2PO4 in 25% ACN, pH2.7) and loaded onto a 4.6 × 250 mm Ultremex SCX column containing 5-μm particles (Phenomenex, CA, USA). The peptides were eluted at a flow rate of 1 mL/min with a gradient of buffer A for 10 min, 5-35% buffer B (25 mM NaH2PO4, 1 M KCl in 25% ACN, pH2.7) for 11 min, 35-80% buffer B for 1 min. The system was then maintained in 80% buffer B for 3 min before equilibrating with buffer A for 10 min prior to the next injection. Elution was monitored by measuring absorbance at 214 nm, and fractions collected every 1 min. The eluted peptides were pooled as 10 fractions, desalted by Strata X C18 column (Phenomenex, CA, USA) and vacuum-dried.

### LC-ESI-MS/MS analysis by LTQ-Orbitrap HCD

Each fraction was resuspended in 1 mL of buffer A (2% ACN, 0.1% FA) and centrifuged at 20000 g for 10 min. In each fraction, the final concentration of peptide was about 0.5 μg/ul on average. Ten μl supernatant was loaded on a Shimadzu LC-20 AD nanoHPLC by the autosampler onto a 2 cm C18 trap column (inner diameter 200 μm) and the peptides were eluted onto a resolving 10 cm analytical C18 column (inner diameter 75 μm) made in-house. The samples were loaded at 15 μL/min for 4 min, and then the 44 min gradient was run at 400 μL/min starting from 2 to 35% B (98% ACN, 0.1% FA), followed by 2 min linear gradient to 80%, and maintenance at 80% B for 4 min, and finally returned to 2% in 1 min.

The peptides were subjected to nanoelectrospray ionization followed by tandem mass spectrometry (MS/MS) in an LTQ Orbitrap Velos (Thermo Fisher, MA, USA) coupled online to the HPLC (Shimadzu, Kyoto, Japan). Intact peptides are detected in the Orbitrap at a resolution of 60000. Peptides were selected for MS/MS using high energy collision dissociation (HCD) operating mode with a normalized collision energy setting of 45%; ion fragments were detected in the LTQ. A data-dependent procedure that alternated between one MS scan followed by eight MS/MS scans was applied for the eight most abundant precursor ions above a threshold ion count of 5000 in the MS survey scan with the following Dynamic Exclusion settings: repeat counts, 2; repeat duration, 30s; and exclusion duration, 120 s. The electrospray voltage applied is 1.5 kV. Automatic gain control (AGC) was used to prevent overfilling of the ion trap; 1 × 10^4^ ions were accumulated in the ion trap for generation of HCD spectra. For MS scans, the m/z scan range was 350 to 2000 Da. The experiment was repeated three times, and the results were categorized as 114 and 117 groups.

### Database search and quantification

The MS/MS data were searched against the uniprot_Bovidae (43,204 sequences) database for peptide identification and quantification using the ProteinPilot software 4.0 (Applied Bio-system, USA). The parameters were set as follows: trypsin as enzyme, methylmethanethio sulphonate of cysteines residues as fixed modification. The Paragon Algorithm (Applied Biosystem, USA) followed by the ProGroup Algorithm (Applied Biosystem, USA) were applied to remove redundant hits to determine the target proteins. Other parameters such as fragment ion accuracy, tryptic cleavage specificity, and allowance for number of missed cleavages were provided and processed by ProteinPilot software. Unused Prot-Score >1.3 (95%) as threshold with at least more than one peptide above the 95% confidence was considered as benchmark for protein identification.

### Electrophoresis and Western blotting

Proteins were extracted from six samples including three healthy and three mastitis-infected mammary gland tissues of Chinese Holstein cows for Western blot analysis. Pre-stained molecular weight standards (Thermo, USA) were included on all gels as a reference. Samples were separated by sodium dodecyl sulfate-polyacrylamide gel electrophoresis (SDS-PAGE, Beyotime, China) on 5% and 12% polyacrylamide gels, and subsequently transferred to polyvinylidene difluoride (PVDF) membrane (Millipore, USA). Following blocking with blocking buffer (Beyotime, China) for 1 h at RT, western blots were probed with rabbit polyclonal Collagen I (Abcam, 1:1000 dilution) and mouse monoclonal β-actin primary antibody (Beyotime, 1:1000 dilution), followed by Horseradish Peroxidase (HRP) labeled goat anti-rabbit IgG (Beyotime, 1:1000 dilution) secondary antibody. The goat polyclonal to mouse ITIH4 (synthetic peptide: KPEGQEQFQVAEK; ab118283) was used as the primary anti-body for the bovine ITIH4 protein of WB. The blots were developed using a Horseradish Peroxidase Color Development Kit (DAB, Beyotime, Nantong, China). Image analysis was performed with Quantity one (Bio-Rad, CA, USA).

### Immunohistochemistry (IHC)

Mammary gland tissues from healthy and mastitis-infected cows were fixed in 4% paraformaldehyde PFA, and then all tissues were embedded in paraffin and sectioned for IHC test as described previously [47]. Briefly, a total of 1 L deionized water and 10 mL citrate buffer solution were used to rehabilitate antigen and washed with 0.01 M PBS (PH 7.4) twice per 3 min. Then, immunoreaction slides were deparaffinaged and hydrated. The slides were blocked with endogenous peroxydase for 10 min and subsequently washed with PBS and incubated with rabbit polyclonal Collagen I (1:100; Abcam, HK, China) and goat polyclonal to ITIH4 (1:100; Abcam, HK, China) for 60 min at RT. After washing with PBS, the slides were incubated with anti-mouse secondary antibody for 10 min at RT. Next, the antibody was visualized with 0.6 mg/mL 3,3′-diaminobenzidine tetrachloride (DAB, Cwbiochem, Beijing, China) Horseradish Peroxidase color development kit (Cwbiochem, Beijing, China) for brown staining under the microscope (Leica LB30T, Germany) according to the manufacturer’s instruction. Finally, the slides were re-stained with hematoxylin (Cwbiochem, Beijing, China) and dried again. Images were captured using a digital camera (Leica, Wetzlar, Germany).

### Bioinformatics

The cellular component (CC), molecular function (MF) and biological process (BP) of the proteins identified by iTRAQ were annotated by the Blast2GO software (http://www.blast2go.org/); GO functional classification and enrichment analysis were also performed to identify GO terms that were significantly enriched in differentially expressed proteins using DAVID analysis (http://david.abcc.ncifcrf.gov/).

**Figure 3:**
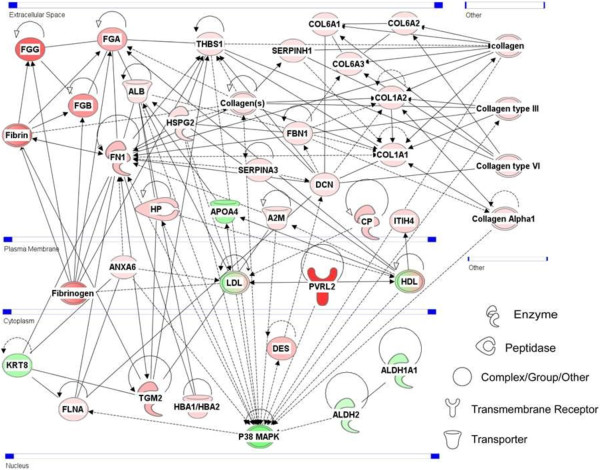
**The most likely IPA network that depict genes related to organismal injury and abnormalities, dermatological diseases and conditions, hereditary disorder (score 68).** The network displayed proteins as nodes having different shapes that represent the functional class of the proteins and the relationships between the nodes as edges (lines). The diagrams show direct (solid lines) and indirect (dashed lines) interactions between proteins. Red and green shading denotes proteins increased and decreased in expression, respectively, and the intensity of the colour indicates the degree of modulation.

We used two different bioinformatic tools to examine the functional relationships among the differentially expressed proteins (DEP): the Dynamic Impact Approach (DIA) and the Ingenuity Pathway Analysis tool version 8.0 (IPA; Ingenuity® Systems, Inc, Redwood City, CA, http://www.ingenuity.com). Canonical pathway and regulatory network analyses were computed with IPA, using as a reference set the Ingenuity Pathways Knowledge Base. To identify the cellular mechanisms most related to mastitis, we searched the Ingenuity Pathways Knowledge Base for networks of interconnected modulated proteins, and for over-represented pathways. IPA is a software created to identify the most significant biological functions, canonical pathways, and networks embedded in a protein/gene set. The list was then uploaded into the IPA software that mapped and annotated 54 proteins, all involved in 3 main networks. IPA ranked networks depending on the reliability of the microarray results on the basis of prior published data. The statistical likelihood (Score) was used to rank the networks and ranged between 65 and 2 (Table 3 and Figure 3).

**Table 3:** **Networks ranking with relative molecules symbols list, score value, number of molecules belonging to the dataset (Focus molecules) and top function for each network**

ID	Molecules in network	Score	Focus molecules	Top functions
1	A2M, ALB, ALDH2, ALDH1A1, Alp, Alpha catenin, ANXA6, Ap1, APOA4, calpain, chymotrypsin, COL1A1, COL1A2, COL6A1, COL6A2, COL6A3, collagen, Collagen Alpha1, Collagen type I, Collagen type III, Collagen type IV, Collagen type VI, Collagen(s), CP, DCN, DES, elastase, ERK1/2, FBN1, FGA, FGB, FGG, Fibrin, Fibrinogen, FLNA, FN1, Focal adhesion kinase, GPIIB-IIIA, Growth hormone, HBA1/HBA2, HDL, HP, HSPG2, IL1, Immunoglobulin, Integrin, ITIH4, KRT8, Laminin, Ldh, LDL, Mmp, P38 MAPK, Pak, Pdgf (complex),PDGF BB, Pka, Rock, S100A9, S100A12, SERPINA3, SERPINH1, Smad, Stat3-Stat3, T3-TR-RXR, Tgf beta, TGM2, THBS1, trypsin, Vegf	68	31	Organismal Injury and Abnormalities, Dermatological Diseases and Conditions, Hereditary Disorder
2	Actin, ADCY, ADM, Akt, Alpha Actinin, ANGPTL1, caspase, CD3, CD27, CD226, CSK, Cytokeratin, D-mannose, ERK, F Actin, Fascin, FKBP1A, GCNT1, GRB7, HBB, hemoglobin, HIST1H1C, Histone h3, Ige, Igf, IgG, IL12 (complex), IL17R, Il3r, Insulin, ITGB8, Jnk, KRT19, L-isoleucine, LCN2, LCP1, LYPLA1, MAPK13, Mapk, MAZ, MB, MYBPH, MYH9, MYH10, MYL1, MYL4, MYL6, MYO15A, Myosin, NEB, NFkB (complex), PER1, PI3, PI3K (complex), PIGR, Pkc(s), PLA2R1, PTPRCAP, PVRL2, Rac, Ras, S100, S100A8, S100A12, SLC5A3, SORBS1, Sos, TCR, TLR2/TLR4, TMPRSS6	25	14	Lymphoid Tissue Structure and Development, Organ Morphology, Cancer
3	AKT1, ALDH1B1, ATP5J2, Casein, COPS8, CORO1B, CPB2, Csn1s1, DAZAP1, DDX18, DIMT1, Fascin, FGA, GAR1, GLUD1, GPI, HNRNPA1L2, Igf, Il3r, IPO4, ITGAX, ITGB8, KIAA1524, KIDINS220, LAP3, LCP1, MDN1, MMP16, MRPS34, MT-CYB, MTHFR, MTM1, MYBPH, MYC, MYL1, NDUFA5, NDUFS2, NOP58, NUAK1, NUP205, PDCD11, PELI2, PGS1, PI4KA, PPA1, PVRL2, SAP130, SEC22B, SLC1A4, SLC20A1, SLC25A1, SLC40A1, SORBS1, SRM, ST6GAL1, Thbs1, THBS2, TIA1, TIAL1, TNF, Top2, TRAP1, UBC, UNC5B, UQCR10, UQCR11, UQCRB, UQCRFS1, Wap, YARS	12	8	Cell Cycle, Cardiovascular Disease, Cell Morphology
4	CLDN24,cldn	2	1

To enhance the biological interpretation of the results, we used the DIA with the 55 differentially expressed proteins identified, considering in the analysis: KEGG pathways, each of the three main GO categories (BP, MF and CC) and, for predicting the presence of domains and important sites within proteins (INTERPRO).

For DIA analysis, information from the freely-available online databases KEGG and DAVID (v6.7) was considered. A list of protein or gene identifiers (Entrez Gene IDs) from *Bos taurus* was uploaded all at once to extract and summarize functional annotations associated with groups of proteins or with each individual protein. Details of the DIA approach and its validation have been reported previously [15]. The direction of the impact (“flux”) was calculated according to our published procedure [48], considering negative flux values as inhibited or downregulated and positive flux values as activated or upregulated. The impact value determines the biological significance of the change on a pathway and/or function by a treatment and/or change in physiological state. For this study, due to the abundance of significant GO terms and KEGG pathways from the DIA output, we considered as significant or more relevant from a biological standpoint those with calculated impact values above 30% of the total impact value of the top-impacted pathways.

### Statistical analysis

In the iTRAQ analysis, the relative expression of proteins was based on the ratio of the reporter ions of the peptides (114:117) using the Mascot software. We adopted the fold change to compare with the differentially expressed proteins. *T*-test was used to analyze the p-value of the value of log_2_ (114/117). Moreover, the protein with the fold change cutoff ratio < 0.66 or >1.50 as well as the p value less than 0.05 was designated the differential expression protein. In the WB analysis, the Student’s *t*-test was used to compare means between two groups. Statistical analyses were conducted with SPSS 11.0 (SPSS, Chicago, IL, USA), and a two-tailed p < 0.05 was considered significant.

## Electronic supplementary material

Additional file 1: **Summaries of peptide and protein identification.** (ZIP 4 MB)

Additional file 2: Table S1: KEGG pathway analysis of the expressed proteins in mammary gland of cows by DAVID. **Table S2**: GO term of differential proteins for BP by Blast2GO. **Table S3**: Pathway analysis of differential proteins by Blast2GO. (XLS 562 KB)

Additional file 3: Figure S1: GO terms of differentially expressed proteins for CC by Blast2GO. (PDF 138 KB)

Additional file 4: Figure S2: GO terms of differentially expressed proteins for MF by Blast2GO. (PDF 120 KB)

Additional file 5: Figure S3: GO terms of differentially expressed proteins for BF by Blast2GO. (PDF 426 KB)

Additional file 6: **DIA analysis of expressed proteins in mammary gland tissues.** Biological Process (BP), Molecular Function (MF), Cellular Component (CC) or Interpro (IPRO) related to those pathways for mastitic vs. healthy cows. Flux represent the direction of each pathway (green color = inhibition, yellow color = stable, red color = activation with different color intensities according with the level of up-regulation or down-regulation). Blue lines show the impact of each GO Term. (XLSX 354 KB)
