# Supplementary figures and images for: iTRAQ-proteomics and bioinformatics analyses of mammary tissue from cows with clinical mastitis due to natural infection with Staphylococci aureus
Source: BMC Genomics. 2014 Oct 2;15(1):839. doi: 10.1186/1471-2164-15-839 (PMC4198675; doi:10.1186/1471-2164-15-839)

<=1e-10
1e-10 to 1e-8
1e-8 to 1e-6
1e-6 to 1e-4
1e-4 to 1e-2
>0.01

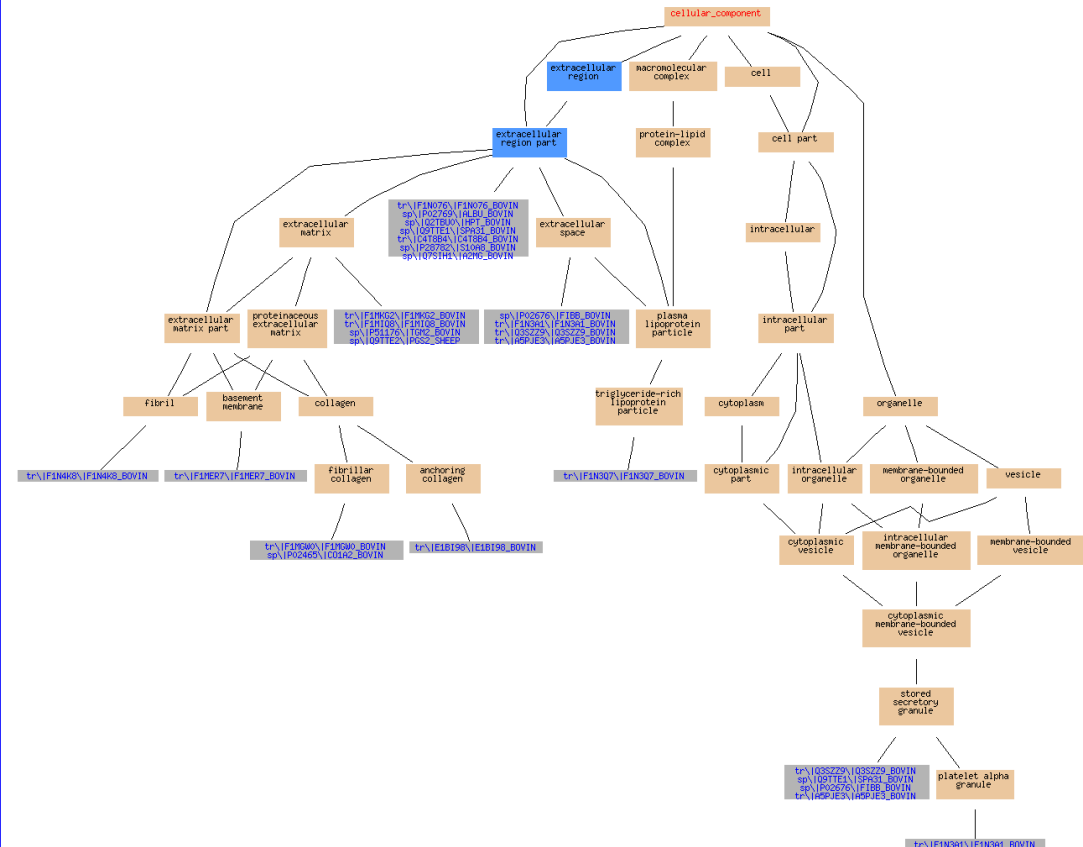

$<10^{-10}$     $10^{-10}$  to  $10^{-8}$     $10^{-8}$  to  $10^{-6}$     $10^{-6}$  to  $10^{-4}$     $10^{-4}$  to  $10^{-2}$     $>0.01$

Supplement: Supplementary file 3 — Additional file 3: Figure S1: GO terms of differentially expressed proteins for CC by Blast2GO. (PDF 138 KB) [file 12864_2014_6543_MOESM3_ESM.pdf]

pvalue:

<=1e-10
1e-10 to 1e-8
1e-8 to 1e-6
1e-6 to 1e-4
1e-4 to 1e-2
>0.01

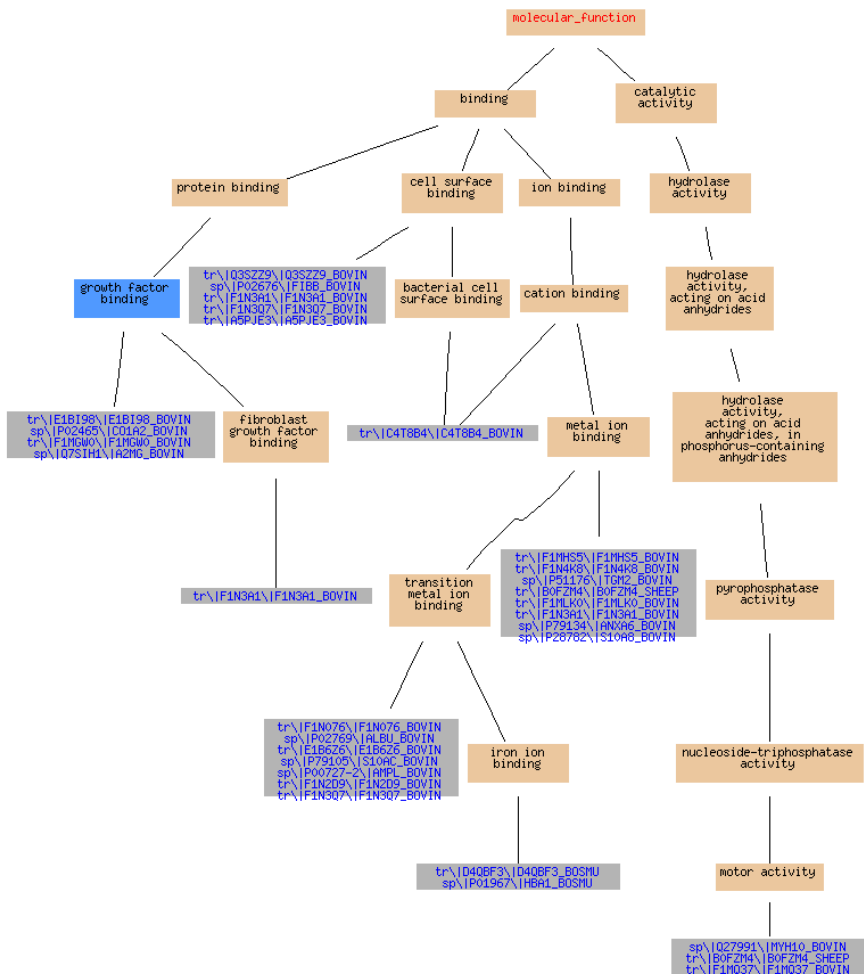

pvalue:

<=1e-10
1e-10 to 1e-8
1e-8 to 1e-6
1e-6 to 1e-4
1e-4 to 1e-2
>0.01

Supplement: Supplementary file 4 — Additional file 4: Figure S2: GO terms of differentially expressed proteins for MF by Blast2GO. (PDF 120 KB) [file 12864_2014_6543_MOESM4_ESM.pdf]

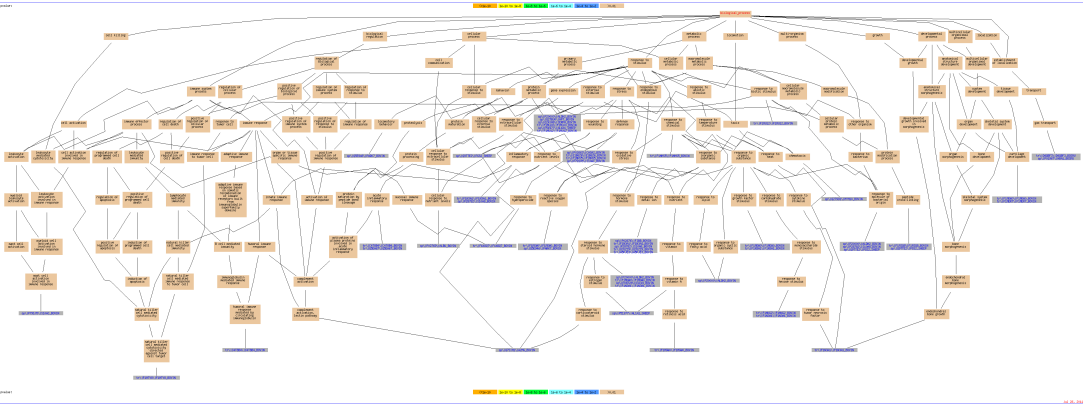

Supplement: Supplementary file 5 — Additional file 5: Figure S3: GO terms of differentially expressed proteins for BF by Blast2GO. (PDF 426 KB) [file 12864_2014_6543_MOESM5_ESM.pdf]
